# LINC00885 promotes cervical cancer progression through sponging miR-3150b-3p and upregulating BAZ2A

**DOI:** 10.1186/s13062-021-00314-6

**Published:** 2022-01-10

**Authors:** Yeling Liu, Jingrui Chen, Lizhong Zhou, Chunhua Yin

**Affiliations:** 1 Anatomy and Pathology Department, Nanchang Medical College, Nanchang, Jiangxi China; 2 School of Clinical Medicine, Nanchang University, Nanchang, Jiangxi China; 3 Department of Obstetrics and Gynecology, Yushan People’s Hospital, Shangrao, Jiangxi China; 4 Department of Obstetrics and Gynecology, The First Affiliated Hospital of Nanchang University, No. 17 Yong Wai Main Street, Nanchang, Jiangxi China

**Keywords:** LINC00885, miR-3150b-3p, BAZ2A, Cervical cancer

## Abstract

**Background:**

Cervical cancer (CC) is one of the most common malignancies affecting female worldwide. Long non-coding RNAs (lncRNAs) are increasingly indicated as crucial participants and promising therapeutic targets in human cancers. The main objective of this study was to explore the functions and mechanism of LINC00885 in CC.

**Methods:**

RT-qPCR and western blot were used to detect RNA and protein levels. Functional and mechanism assays were respectively done for the analysis of cell behaviors and molecular interplays.

**Results:**

Long intergenic non-coding RNA 885 (LINC00885) was discovered to be upregulated in CC tissues and cell lines through bioinformatics analysis and RT-qPCR. Overexpression of LINC00885 promoted proliferation and inhibited apoptosis, whereas its silence exerted opposite effects. The cytoplasmic localization of LINC00885 was ascertained and furthermore, LINC00885 competitively bound with miR-3150b-3p to upregulate BAZ2A expression in CC cells. Rescue assays confirmed that LINC00885 regulated CC proliferation and apoptosis through miR-3150b-3p/BAZ2A axis. Finally, we confirmed that LINC00885 aggravated tumor growth through animal experiments.

**Conclusions:**

LINC00885 exerted oncogenic function in CC via regulating miR-3150b-3p/BAZ2A axis. These findings suggested LINC00885 might serve as a potential promising therapeutic target for CC patients.

**Supplementary Information:**

The online version contains supplementary material available at 10.1186/s13062-021-00314-6.

## Background

Cervical cancer (CC), one of leading causes of cancer death, has approximately 527,600 cases of diagnosis and 265,700 cases of death globally in 2012 [1]. Despite that past years have witnessed the improvement of therapeutic regimes and that CC patients at early stages could be cured via surgery [2], the survival rate of CC patients at advanced metastatic stage remains low, which is only approximately 15% [3–6]. Hence, efficient molecular targets are needed to be identified to further develop the therapies for CC patients.

Long non-coding RNAs (lncRNAs), as non-protein coding RNA molecules with more than 200 bases in length [7, 8], have been demonstrated to be key participators in diverse pathophysiological processes, including cancer progression [9–11]. The dysregulation and function of lncRNAs have been revealed in numerous cancers, indicating that lncRNAs could be promising prognostic biomarkers in cancers [12–14]. To date, many lncRNAs have been proven to exert regulatory functions in cervical cancer [15, 16]. Present study identified long intergenic non-coding RNA 885 (LINC00885) to be upregulated in cervical cancer. LINC00885 has been proven to correlate with GATA3 in bladder cancer [17]. Moreover, the association between LINC00885 and early stage breast cancer progression has also been validated [18]. Therefore, the functions of LINC00885 and the underlying regulation mechanisms in other malignancies including CC deserve further exploration.

Previous findings have suggested that lncRNAs regulate gene expressions through multiple manners. The mechanism by which lncRNAs modulate gene expression varies according to the cellular localization [19]. In cytoplasm, lncRNAs can regulate gene expression by performing as competitive endogenous RNAs (ceRNA) [20]. By sponging miRNAs, lncRNAs prevent microRNAs (miRNAs) from targeting downstream genes at the 3' untranslated region (3'UTR) and resulting in variation in mRNA expression [21]. Also, studies have shown that lncRNAs work through ceRNA network to regulate cervical cancer progression [22, 23]. MiR-3150b-3p has been predicted by us to be a target for LINC00885, but never has it been explored by previous study before in any cancer, nor has it been related to LINC00885 previously.

Bromodomain adjacent to zinc finger domain 2A (BAZ2A), also named as TIP5, is the component of nucleolar remodeling complex (NoRC) belonging to imitation switch chromatin remodeling complexes (ISWI) [24–26]. BAZ2A has been reported to be responsible for epigenetic regulation on recombinant RNA genes [27, 28]. Additionally, BAZ2A has been shown by several studies to predict recurrence of prostate cancer and regulate hepatocellular cancer [29, 30]. However, the function of BAZ2A in cervical cancer and its correlation with LINC00885 or miR-3150b-3p have not been explored yet.

Our study is to probe into the function and modulatory mechanism of LINC00885 in CC.

## Results

### LINC00885 was upregulated in CC and predicted poor prognosis

**Fig. 1:**
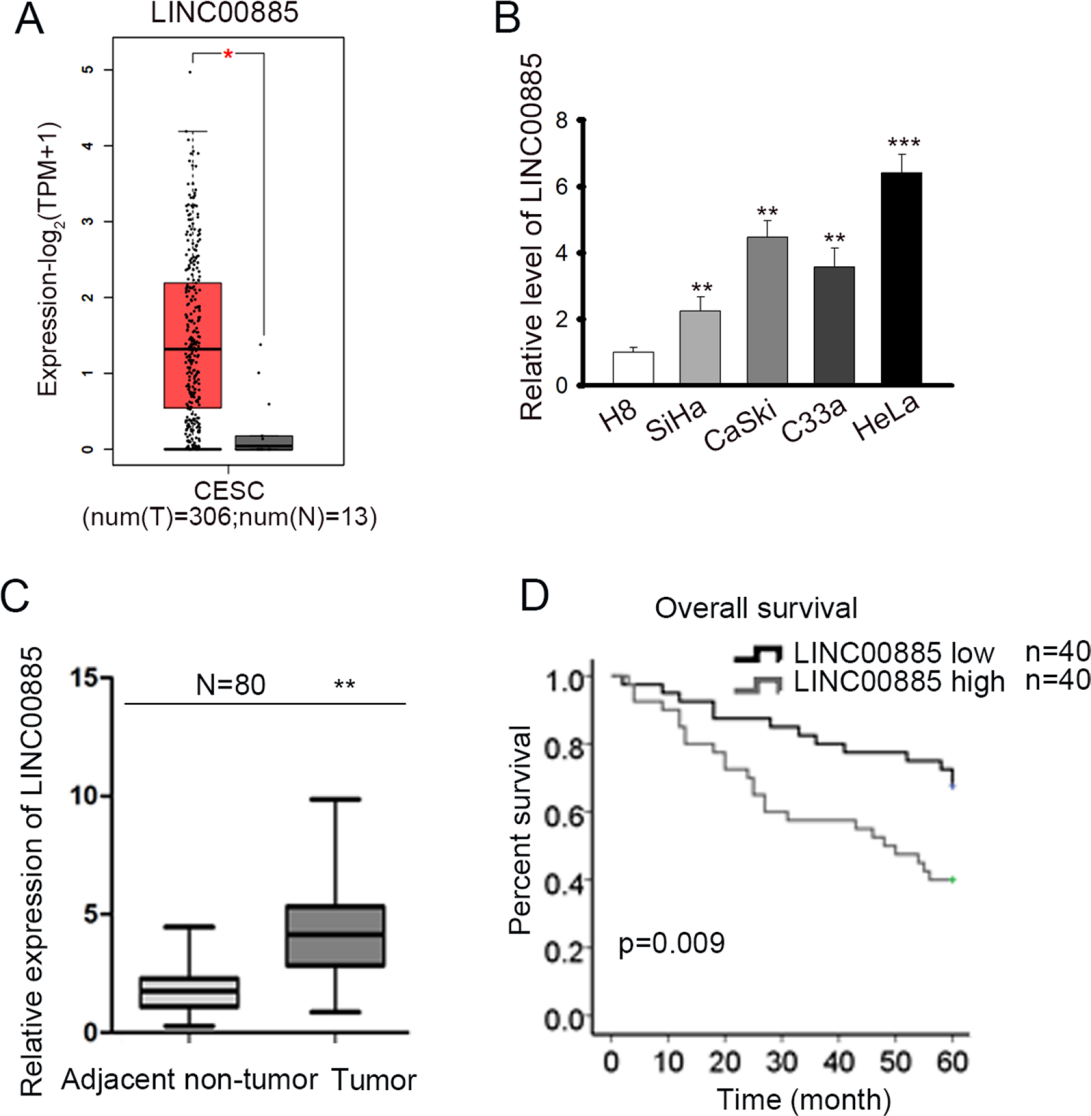
LINC00885 was upregulated in CC and predicted poor prognosis. **A** Bioinformatics tool (GEPIA) was applied for analyzing the expressions of LINC00885 in CESC tissues and normal tissues. **B** RT-qPCR was done for the analysis of LINC00885 expression in four CC cell lines (SiHa, CaSki, C33a, and HeLa) and cervical epithelial immortalized cell line (H8). **C** RT-qPCR was done for the measurement of LINC00885 expression in 80 CC samples and the matched para-cancerous samples. **D** Kaplan–Meier analysis and log-rank test confirmed the prognostic significance of LINC00885 expression in CC patients. ^*^P < 0.05, ^**^P < 0.01, ^***^P < 0.001

To explore the functions of LINC00885 in CC, the expression pattern of LINC00885 in cervical squamous cell carcinoma (CESC) samples was firstly obtained from GEPIA database (http://gepia.cancer-pku.cn/index.html), a comprehensive resource for systematic analysis of gene expression [31]. In addition to differential thresholds (|log2FC| Cutoff: 1; p-value Cutoff: 0.01), the method for differential analysis based on the selected datasets (TCGA tumors vs TCGA normal + GTEx normal) was one-way ANOVA by calculating differential expression. It was found that LINC00885 presented significantly higher level in CESC samples (Fig. 1a), indicating that LINC00885 might be associated with CC progression. Therefore, we focused on the exploration of LINC00885 functions in CC. By detecting LINC00885 expression in cell lines through Reverse transcription-quantitative polymerase chain reaction (RT-qPCR), we found that LINC00885 expression was much higher in CC cell lines than in cervical epithelial immortalized cells (H8), and among all CC cell lines, HeLa cells presented the highest LINC00885 level and SiHa the lowest (Fig. 1b). Also, we confirmed that LINC00885 was upregulated in 80 CC tissues versus the paired adjacent non-tumor tissues (Fig. 1c), and higher expression of LINC00885 predicted lower overall survival of CC patients (Fig. 1d). These results suggested that LINC00885 was upregulated in CC and predicted poor prognosis.

### LINC00885 facilitated proliferation and retarded apoptosis of CC cells

**Fig. 2:**
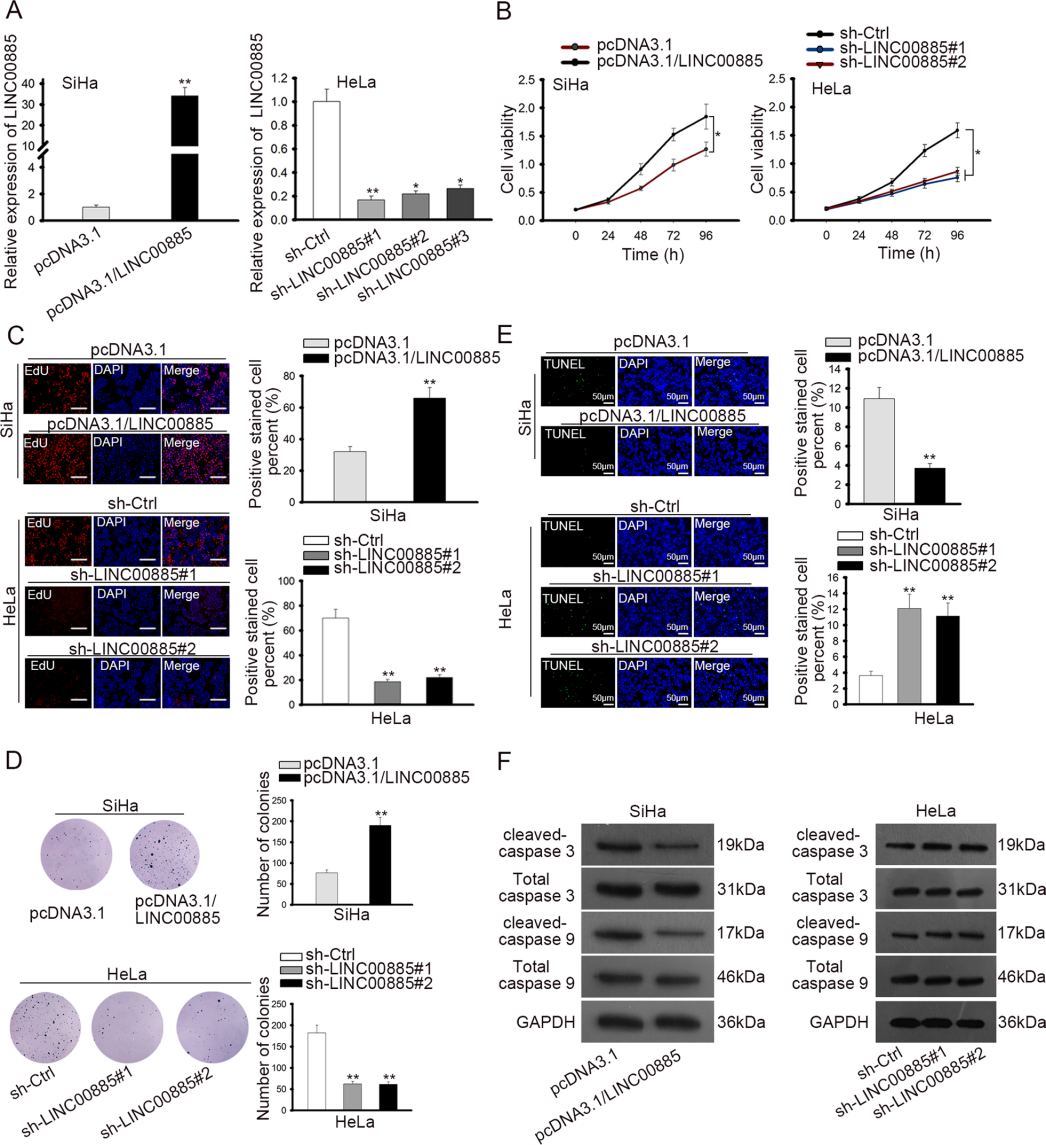
LINC00885 facilitated proliferation and retarded apoptosis of CC cells. **A** RT-qPCR analysis was done for the detection of LINC00885 in pcDNA3.1/LINC00885 transfected SiHa cells and sh-LINC00885#1/2/3 transfected HeLa cells. **B**–**D** CCK-8, EdU, and colony formation assays were used to detect proliferation of SiHa and HeLa cells in response to LINC00885 overexpression and knockdown. **E**–**F** Apoptosis of SiHa and HeLa cells was detected by TUNEL assay and by examining the apoptosis-related genes (cleaved- and total caspase 3/9) via western blot analysis. *P < 0.05, **P < 0.01

Next, to probe into the biological role of LINC00885 in CC cells, we overexpressed LINC00885 with pcDNA3.1/LINC00885 in SiHa cells and silenced LINC00885 with sh-LINC00885#1/2/3 in HeLa cells, and the transfection efficiency was confirmed to be high by RT-qPCR results (Fig. 2a). In following experiments, sh-LINC00885#1/2 and pcDNA3.1/LINC00885 were applied. Cell counting kit-8 (CCK-8) and 5-Ethynyl-2′-deoxyuridine (EdU) assay results demonstrated that proliferation of SiHa cells with LINC00885 overexpression was promoted compared with control group, whereas proliferation of HeLa cells with LINC00885 silence was hampered versus control (Fig. 2b, c). Additionally, colony formation assay showed that SiHa cells with LINC00885 overexpression generated more colonies than control cells, whereas HeLa cells with LINC00885 knockdown generated fewer colonies than control cells (Fig. 2d). Terminal deoxynucleotidyl transferase-mediated dUTP nick end labeling (TUNEL) assay showed that overexpressing LINC00885 attenuated cell apoptosis, whereas silencing LINC00885 facilitated cell apoptosis (Fig. 2e). Additionally, expressions of apoptotic genes (cleaved-caspase 3, total caspase 3, cleaved-caspase 9 and total caspase 9) were evaluated by western blot analysis. Consequently, protein expressions of cleaved-caspase 3 and cleaved-caspase 9 decreased on account of the elevation in LINC00885 expression, whereas protein expressions of cleaved-caspase 3 and cleaved-caspase 9 increased in response to LINC00885 knockdown (Fig. 2f). In sum, these data suggested that LINC00885 facilitated proliferation and retarded apoptosis of CC cells.

### MiR-3150b-3p was the downstream of LINC00885 in CC

**Fig. 3:**
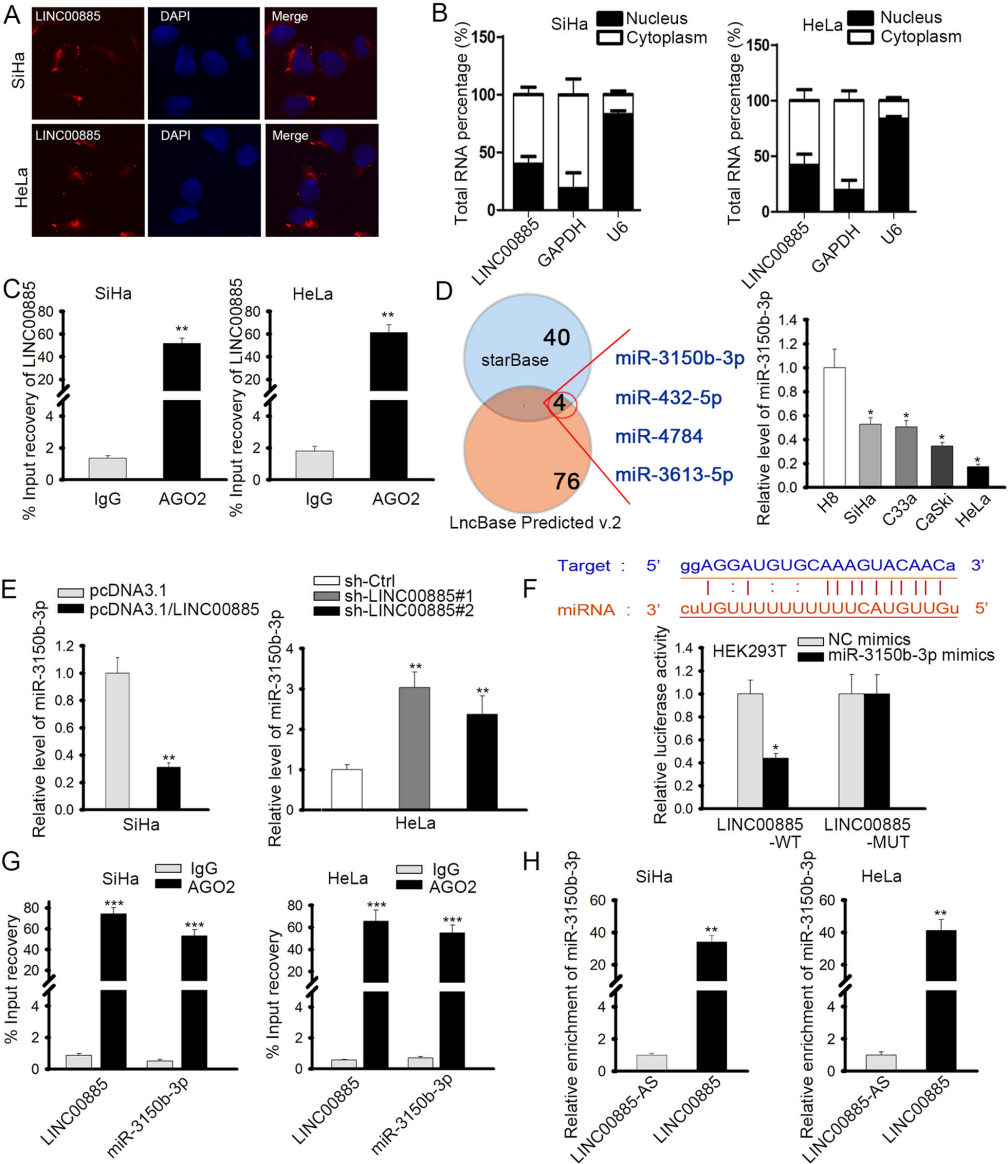
LINC00885 modulated miR-3150b-3p in CC cells. **A**, **B** FISH and subcellular fractionation were used to determine the localization of LINC00885 in SiHa and HeLa cells. **C** RIP assay showed that LINC00885 could be immunoprecipitated in Ago2 antibody. **D** Based on prediction on starBase and LncBase Predicted v.2, 4 miRNAs (miR-3150b-3p, miR-432-5p, miR-4784, and miR-3613-5p) might bind to LINC00885 (left). RT-qPCR analysis was done for the detection of miR-3150b-3p in different cell lines (right). **E** RT-qPCR analysis was used to examine the expression of miR-3150b-3p in SiHa cells with LINC00885 overexpression and in HeLa cells with LINC00885 silence. **F** After prediction of the binding sites between LINC00885 and miR-3150b-3p, luciferase reporter assay was done in HEK293T cells for the evaluation of the interaction between miR-3150b-3p and LINC00885. **G**, **H** RIP assay and pulldown assay were used to evaluate the interaction between LINC00885 and miR-3150b-3p. *P < 0.05, **P < 0.01, ***P < 0.001

Then, the specific regulation mechanism of LINC00885 in CC cells was taken into consideration. Since lncRNAs exerted their functions in cancers through regulating certain gene expressions, and their regulatory manners vary according to the subcellular location [19], we first detected the localization of LINC00885 in CC cells. Results from fluorescence in situ hybridization (FISH) and subcellular fractionation assays validated the location of LINC00885 expression was mainly in cytoplasm of CC cells (Fig. 3a, b). In cytoplasm, lncRNAs could act as ceRNAs by sponging miRNAs [20], so we speculated that LINC00885 might participate in ceRNA network in CC. To validate our hypothesis, we first detected whether LINC00885 existed in RNA-induced silencing complex (RISC). RNA immunoprecipitation (RIP) assay confirmed that LINC00885 could be immunoprecipitated in anti-Ago2, suggesting that LINC00885 could act as a ceRNA (Fig. 3c). Therefore, we further tried to identify the miRNAs potentially interacting with LINC00885 in CC. Combining the prediction results of starBase (http://starbase.sysu.edu.cn/index.php) and LncBase Predicted v.2 (http://carolina.imis.athena-innovation.gr/diana_tools/web/index.php?r=lncbasev2%2Findex-predicted), we found four candidate miRNAs (miR-3613-5p, miR-432-5p, miR-4784, and miR-3150b-3p) targeted by LINC00885 (Fig. 3d, left). To figure out which miRNA was actually involved in CC, we detected their expressions in cell lines. It turned out that only miR-3150b-3p was downregulated in four CC cell lines (Fig. 3d, right; Additional file 1: Fig. S1A), suggesting that miR-3150b-3p might participate in the ceRNA mechanism regarding LINC00885 in CC cells.

Thereafter, we further investigated the interaction between LINC00885 and miR-3150b-3p in CC cells. RT-qPCR results demonstrated that LINC00885 overexpression could reduce miR-3150b-3p expression, whereas LINC00885 silence could elevate the expression of miR-3150b-3p (Fig. 3e). The binding site between LINC00885 and miR-3150b-3p were presented, and luciferase reporter assay showed that overexpressing miR-3150b-3p attenuated the luciferase activity of LINC00885-WT but had no impact on LINC00885-MUT (Fig. 3f). RIP assay illustrated that miR-3150b-3p and LINC00885 could be co-immunoprecipitated in anti-Ago2, further confirming the interaction between LINC00885 and miR-3150b-3p (Fig. 3g). In addition, RNA pulldown showed that miR-3150b-3p could be pulled down only by LINC00885 probe rather than antisense LINC00885 probe (Fig. 3h). Together, these results implied that miR-3150b-3p was the downstream of LINC00885 in CC cells.

### LINC00885 regulated CC cell proliferation and apoptosis through miR-3150b-3p

**Fig. 4:**
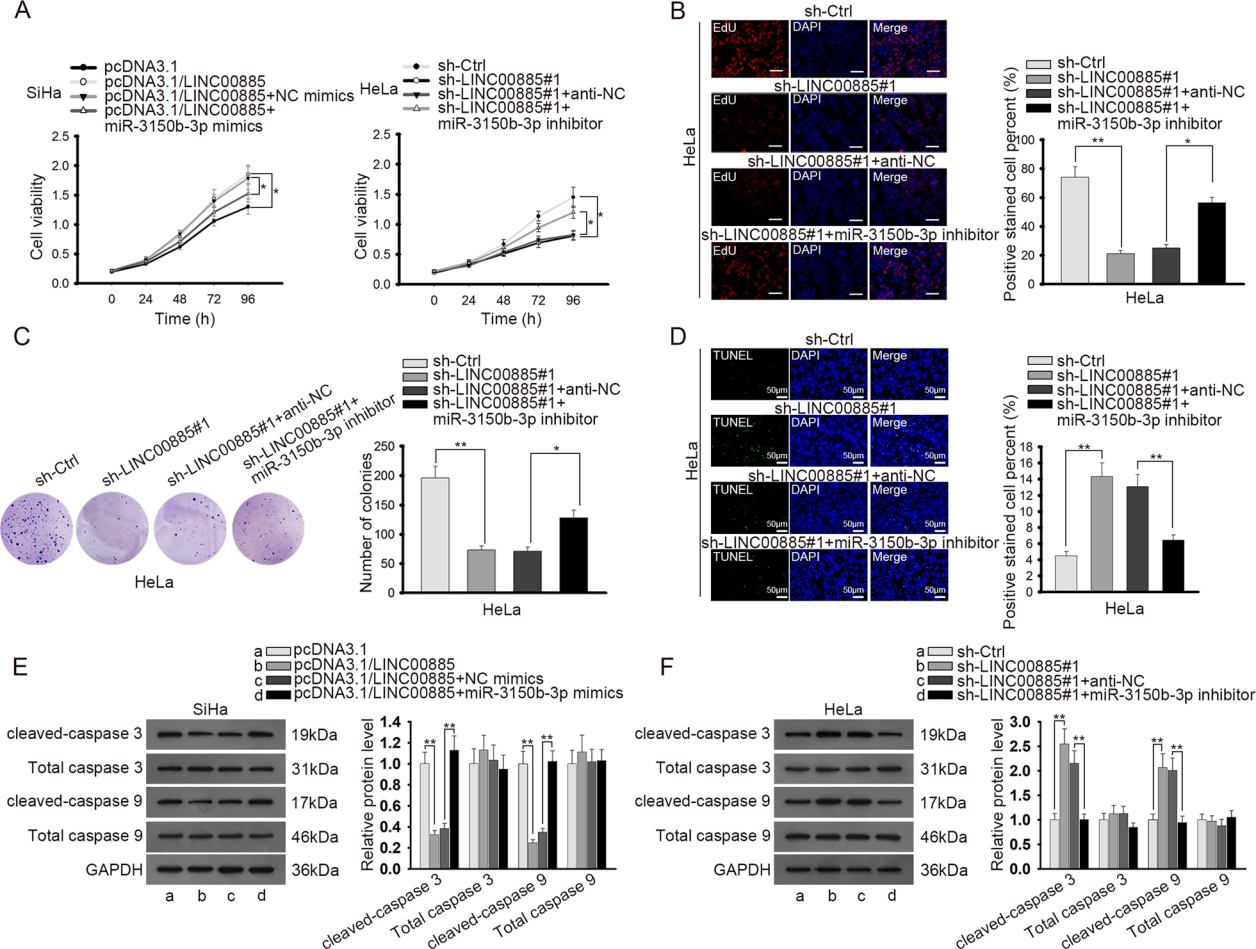
LINC00885 regulated proliferation and apoptosis of CC cells through modulating miR-3150b-3p expression. SiHa cells were transfected with pcDNA3.1, pcDNA3.1/LINC00885, pcDNA3.1/LINC00885 + NC mimics, and pcDNA3.1/LINC00885 + miR-3150b-3p mimics respectively. HeLa cells were transfected with sh-NC, sh-LINC00885#1, sh-LINC00885#1 + anti-NC, or sh-LINC00885#1 + miR-3150b-3p inhibitor. **A** CCK-8 was used to examine proliferation of SiHa and HeLa cells transfected with indicated plasmids. **B**–**C** EdU and colony formation assays were used to examine proliferation of HeLa cells under indicated conditions. **D** TUNEL assay was used to evaluate apoptosis of HeLa cells in different groups. **E** Western blot analysis was done for the measurement of apoptosis-related genes in SiHa and HeLa cells with the transfection of indicated plasmids. *P < 0.05, **P < 0.01

Then, we designed rescue assays to investigate whether LINC00885 regulated CC cell proliferation and apoptosis through miR-3150b-3p. We discovered through CCK-8 that overexpression of miR-3150b-3p abrogated the promoting effect of LINC00885 overexpression on cell proliferation, and that miR-3150b-3p silence counteracted the prohibitive effect of LINC00885 depletion on cell proliferation (Fig. 4a). EdU assay showed that silencing LINC00885 reduced the proliferative CC cells, and such effect was countervailed by inhibiting miR-3150b-3p (Fig. 4b). Colony formation assay showed that number of cell colonies decreased in response to LINC00885 silence, and the trend was reversed by miR-3150b-3p inhibition (Fig. 4c). TUNEL assay elucidated that the aggravated cell apoptosis induced by LINC00885 silencing could be reversed by miR-3150b-3p inhibition (Fig. 4d). Also, the western blot results of the apoptotic genes showed that the decreased levels of cleaved-caspase 3 and cleaved-caspase 9 induced by LINC00885 overexpression could be recovered by miR-3150b-3p overexpression, and that the increased levels of cleaved-caspase 3 and cleaved-caspase 9 caused by LINC00885 downregulation could be reversed by miR-3150b-3p inhibition (Fig. 4e). In sum, the above data indicated that LINC00885 regulated CC cell proliferation and apoptosis through miR-3150b-3p.

### LINC00885 upregulated BAZ2A through targeting miR-3150b-3p

**Fig. 5:**
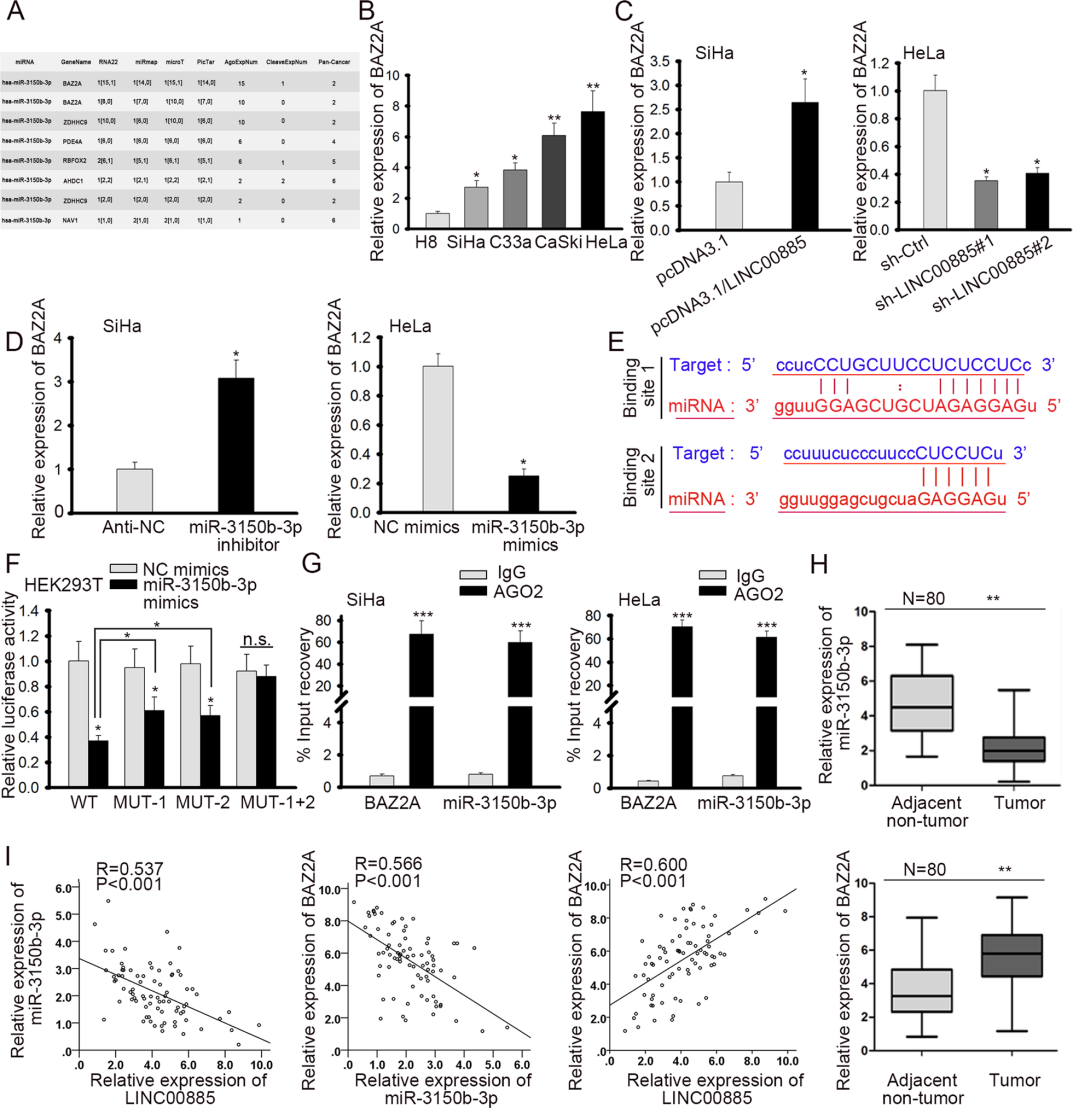
LINC00885 upregulated BAZ2A through regulating miR-3150b-3p. **A** The putative target mRNAs (BAZ2A, ZDHHC9, PDE4A, RBFOX2, AHDC1, and NAV1) for miR-3150b-3p were predicted on RNA22, miRmap, microT, and PicTar databases. **B** RT-qPCR was done for detecting the expression level of BAZ2A in CC cell lines and cervical epithelial immortalized cell line (H8). **C** RT-qPCR was applied for the measurement of BAZ2A expression in SiHa and HeLa cells under the influence of LINC00885 overexpression or knockdown. **D** RT-qPCR analysis was done for the detection of BAZ2A expression in SiHa cells with miR-3150b-3p inhibition and in HeLa cells with miR-3150b-3p overexpression. **E** The binding sequences of miR-3150b-3p on BAZ2A 3’UTR were demonstrated. **F**, **G** Luciferase reporter and RIP assays validated the interaction between miR-3150b-3p and BZA2A. **H** RT-qPCR was applied for the measurement of miR-3150b-3p and BAZ2A in adjacent non-tumor and CC tumor tissues. **I** Spearman’s correlation analysis showed the negative correlation between miR-3150b-3p and LINC00885 or BAZ2A, and the positive correlation between LINC00885 and BAZ2A in CC tissues. *P < 0.05, **P < 0.01, ***P < 0.001, n.s.: no significance

Subsequently, we browsed several online bioinformatics tools to find the target gene for miR-3150b-3p. After analyzing the searching results, we found 6 candidate targets (BAZ2A, ZDHHC9, PDE4A, RBFOX2, AHDC1, and NAV1) of miR-3150b-3p (Fig. 5a). To explore the involvement of these genes in CC, we detected their expressions in cell lines. Results showed that only BAZ2A was upregulated in all four CC cell lines (Fig. 5b, Additional file 1: Fig. S1B). Hence, we selected BAZ2A and further investigated the effect of LINC00885/miR-3150b-3p on BAZ2A expression. RT-qPCR and western blot analyses showed that overexpressing LINC00885 or inhibiting miR-3150b-3p in SiHa cells could augment the mRNA and protein levels of BAZ2A, whereas silencing LINC00885 or elevating miR-3150b-3p in HeLa cells exhibited opposite effects (Fig. 5c, d). Later, we probed into the interaction between miR-3150b-3p and BAZ2A. The two binding sites on BAZ2A 3’UTR for miR-3150b-3p were presented in Fig. 5e. Luciferase reporter assay illustrated that miR-3150b-3p overexpression attenuated the luciferase activity of BAZ2A-WT, and such attenuation could be partly recovered by mutating only site 1 or 2 on BAZ2A 3’UTR. However, miR-3150b-3p had no impact on the luciferase activity of BZA2A-MUT (1 + 2) (Fig. 5f). This result indicated that both two binding sites were responsible for the interaction between miR-3150b-3p and BAZ2A. RIP assay further confirmed the interplay between miR-3150b-3p and BAZ2A (Fig. 5g). Furthermore, we verified that miR-3150b-3p was downregulated, and BAZ2A was upregulated in CC tissues (Fig. 5h). In CC tissues, we validated the negative correlation between miR-3150b-3p and LINC00885 or BAZ2A, and the positive correlation between LINC00885 and BAZ2A (Fig. 5i). Hence, the above results suggested that LINC00885 upregulated BAZ2A through targeting miR-3150b-3p.

### LINC00885/miR-3150b-3p/BAZ2A axis regulated proliferation and apoptosis of CC cells

**Fig. 6:**
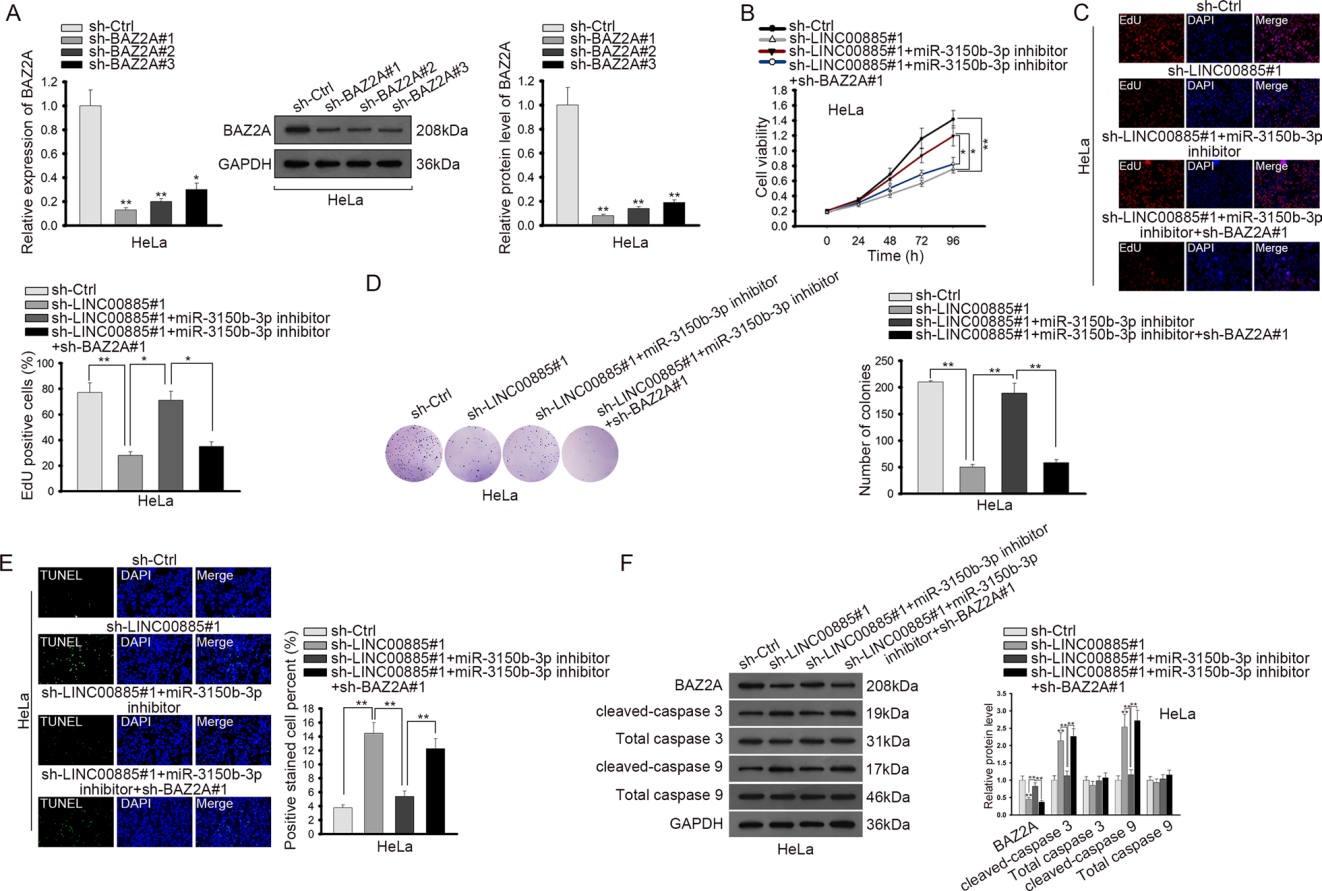
LINC00885/miR-3150b-3p/BAZ2A axis regulated cell proliferation and apoptosis in CC. **A** BAZ2A was silenced by transfection of sh-BZA2A#1/2/3 in HeLa cells as indicated in RT-qPCR and western blot analyses. HeLa cells were transfected with sh-Ctrl, sh-LINC00885#1, sh-LINC00885#1 + miR-3150b-3p inhibitor, or sh-LINC00885#1 + miR-3150b-3p inhibitor + sh-BAZ2A#1 respectively for subsequent assays. **B**–**D** CCK-8, EdU and colony formation assays were done to detect proliferation of HeLa cells transfected with indicated plasmids. **E**–**F** TUNEL assay and western blot analysis of expression levels of apoptosis-related genes (cleaved and total caspase 3 and caspase 9) were done to determine apoptosis of HeLa cells under different transfection conditions. *P < 0.05, **P < 0.01

Thereafter, we conducted rescue assays to detect how LINC00885/miR-3150b-3p/BAZ2A axis functioned in CC. BAZ2A was silenced by three shRNAs in HeLa cells, and the results of RT-qPCR showed that sh-BAZ2A#1 exhibited the highest knockdown efficiency (Fig. 6a). CCK-8 and EdU analyses depicted that inhibiting miR-3150b-3p countervailed the inhibitory effect of LINC00885 silencing on cell proliferation, and such results could be reversed by co-transfection of sh-BAZ2A#1 (Fig. 6b, c). Besides, inhibiting miR-3150b-3p countervailed the inhibitory effect of LINC00885 knockdown on colony formation, and such result could be reversed by silencing BAZ2A (Fig. 6d). TUNEL assay and western blot analysis revealed that the promoting effect of LINC00885 silencing on cell apoptosis and on the expression levels of apoptotic genes (cleaved-caspase 3 and cleaved-caspase 9) could be abrogated by miR-3150b-3p inhibition, and such abrogation could be reversed by co-transfection of sh-BAZ2A#1 (Fig. 6e, f). Therefore, these results suggested that LINC00885/miR-3150b-3p/BAZ2A axis regulated cell proliferation and apoptosis in CC.

### LINC00885 promoted tumor growth in CC in vivo

**Fig. 7:**
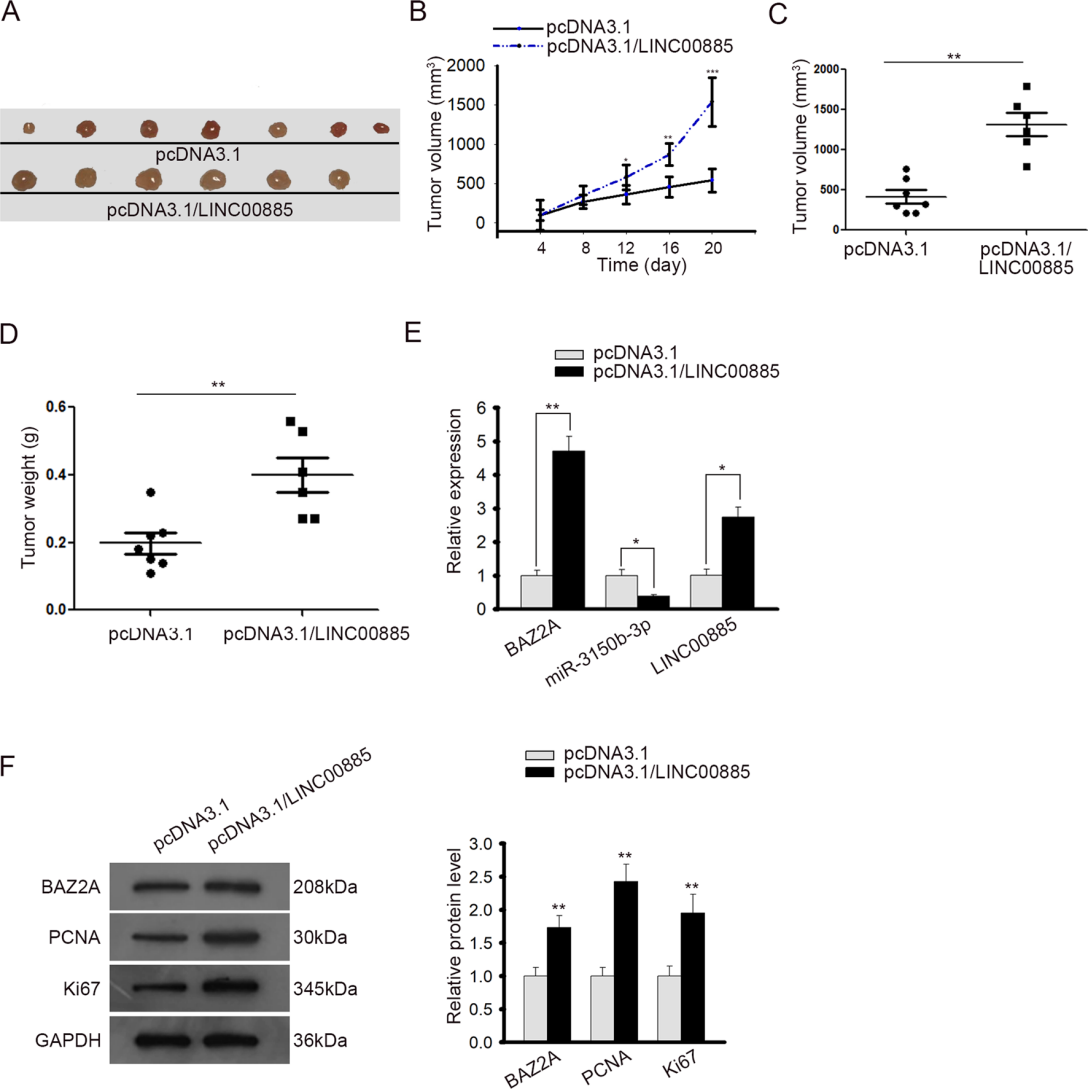
LINC00885 promoted CC tumor growth in vivo. **A** Nude mice were injected with SiHa cells transfected with pcDNA3.1 or pcDNA3.1/LINC00885 for xenografts. The representative pictures of tumors from indicated groups were taken. **B** The tumor growth was monitored every 4 days for consecutive 20 days. **C**, **D** The tumor volume and weight were detected. **E** The expression levels of LINC00885, miR-3150b-3p, and BAZ2A in tumor tissues were measured by RT-qPCR. **F** Western blot analysis was used to detect the expression of BAZ2A, PCNA, and Ki67 in tumors from mice of each group. *P < 0.05, **P < 0.01, ***P < 0.001

Finally, we sought to probe into the influences of LINC00885 on CC progression in animal models. SiHa cells were transfected with pcDNA3.1 or pcDNA3.1/LINC00885, and then were injected into nude mice to generate xenografts. The tumor volume was examined every 4 days. Consequently, we discovered that mice injected with LINC00885 overexpressed SiHa cells generated bigger tumors (Fig. 7a). The overexpression of LINC00885 facilitated tumor growth in vivo (Fig. 7b). Additionally, both the volume and weight of tumor increased in LINC00885 overexpression group (Fig. 7c, d). We also confirmed that overexpression of LINC00885 increased the levels of LINC00885 and BAZ2A, and decreased the level of miR-3150b-3p in vivo (Fig. 7e). Moreover, western blot analysis showed that overexpressing LINC00885 resulted in an augment in the protein levels of BAZ2A and proliferation markers (Ki67 and PCNA) in xenografts (Fig. 7f). To be concluded, these results indicated that LINC00885 promoted CC tumor growth in vivo.

## Discussion

According to statistics, the prognosis of cervical cancer (CC) patients remains unsatisfactory in spite of the improving treatments [3–5]. This indicates an urgent need for the identification of therapeutic targets in CC.

Aberrant expression of lncRNAs in human cancers has been increasingly reported by studies, suggesting lncRNAs as promising prognostic and diagnostic biomarkers in cancers [12–14]. Furthermore, lncRNAs have also been demonstrated to be closely related to the tumor initiation and progression of CC [32, 33]. In current study, we found that LINC00885 was upregulated in CC tissues from GEPIA database and collected patient samples, as well as in CC cell lines, indicating that LINC00885 was implicated in CC progression. Moreover, we revealed that LINC00885 upregulation was associated with poor prognosis of CC patients. Previous study has proven that LINC00885 was related to GATA3 expression in bladder cancer [17]. Moreover, LINC00885 has been validated to play an oncogenic in breast cancer [18]. Consistently, functional assays showed that LINC00885 facilitated proliferation and attenuated apoptosis CC cells, indicating the oncogenic effect of LINC00885 in CC.

Through detecting the localization of LINC00885, we validated its distribution in cytoplasm of CC cells. Considering that the cellular localization of lncRNA could determine their regulatory mechanism in cancers [19], and that cytoplasmic lncRNAs could function as ceRNA in cancers [22, 23], including CC [20], we speculated that LINC008855 could also serve as ceRNA in CC. The immunoprecipitation of LINC00885 in Ago2 antibody suggested that LINC00885 existed in RISC, which meant that LINC00885 might serve as a ceRNA via the competitive binding to miRNA. By searching bioinformatics websites, we identified 4 miRNAs putatively interacting with LINC00885, but only miR-3150b-3p was detected to be downregulated in CC cells, indicating its involvement in LINC00885 modulation mechanism implicated in CC cells. Additionally, miR-3150b-3p was detected to be down-regulated in CC tissues for the first time, and the negative correlation between LINC00885 and miR-3150b-3p was also revealed via bioinformatics analysis and RT-qPCR. Rescue assays suggested that LINC00885 regulated CC cell proliferation and apoptosis through miR-3150b-3p.

After series of experiments, we identified BAZ2A as a target for miR-3150b-3p in CC. BAZ2A is a component in the component of nucleolar remodeling complex (NoRC), responsible for the epigenetic regulation on recombinant RNA genes [27, 28]. Previously, BAZ2A has been reported to be a predictor of prostate cancer recurrence and a participator in hepatocellular cancer [29, 30], indicating the oncogenic role of BAZ2A in cancers. Concordantly, we firstly confirmed the upregulation of BAZ2A in CC, indicating the association of BAZ2A with CC development. Besides, bioinformatics analysis and experimental results showed that LINC00885 positively regulated BAZ2A expression and miR-3150b-3p negatively regulated BAZ2A expression, indicating that BAZ2A was regulated by LINC00885/miR-3150b-3p axis. Based on a recent study, LINC00885 could facilitate CC tumorigenesis by sponging miR-432-5p to up-regulate MACC1 [34]. Results of current rescue assays indicated that LINC00885 regulated CC cell proliferation and apoptosis through miR-3150b-3p/BAZ2A axis. Finally, in vivo assays validated that LINC00885 promoted tumor growth in CC and regulated miR-3150b-3p/BAZ2A axis. Therefore, a novel mechanism of LINC00885 in CC was revealed. Still, larger scale of clinical samples required to be involved in our further study as more convincing evidence.

In conclusion, our study illustrated that LINC00885 facilitated CC cell proliferation and attenuated apoptosis by regulating miR-3150b-3p/BAZ2A axis, indicating LINC00885 as a potential novel prognostic and treatment target in CC.

## Materials and methods

### Tissue collection

80 CC tissues and the matched adjacent non-cancerous tissues were collected from patients at the First Affiliated Hospital of Nanchang University. Permission of this study was given by the Ethics Approval Committee of the First Affiliated Hospital of Nanchang University. The written informed consents had been collected from all patients, who underwent no chemo- or radiotherapy prior to the surgical dissection. After dissection, tissues were instantly snap-frozen in the liquid nitrogen and maintained under −80 °C for subsequent use.

### Cell lines and cell culture

CC cell lines (C33A, HeLa, CaSki, SiHa) and human embryonic kidney cell line 293 T (HEK293T) were bought from BeNa Culture Collection (Beijing, China). The cervical epithelial immortalized cells (H8) were provided by the Cell Resource Database of Chinese Academy of Sciences (Shanghai, China). To culture the cells, Roswell Park Memorial Institute (RPMI)-1640 (Thermo Fisher Scientific, Waltham, MA, USA) was used, with the supplementary of 10% fatal bovine serum (FBS; Wisent, Quebec, Canada), streptomycin (100 µg/mL), and penicillin (100 µg/mL). The incubation atmosphere was humidified, with 95% air and 5% CO_2_ at 37 °C.

### Cell transfection

MiR-3150b-3p inhibitor and miR-3150b-3p mimics (RiboBio, Guangzhou, China) were used for the knockdown or overexpression of miR-3150b-3p, and the negative controls were anti-NC and NC mimics. LINC00885 and BAZ2A knockdown was realized by specific short hairpin RNAs (shRNAs) targeting LINC00885 and BAZ2A, termed sh-LINC00885#1/2/3 and sh-BAZ2A#1/2/3. The scramble shRNAs (shCtrl) were negative controls. The sequences of sh-LINC00885 and sh-BAZ2A, as well as the corresponding sh-Ctrl, were listed in Additional file. The overexpression of LINC00885 was realized through pcDNA3.1/LINC00885 plasmids, with empty vector pcDNA3.1 (GenePharma, Suzhou, China) as negative controls. The cell transfection was accomplished by the utilization of Lipofectamine 2000 (Invitrogen) as required.

### RT-qPCR

To obtain total RNAs from CC tumors or cells, the Trizol reagent (Invitrogen) was utilized. NanoDrop was applied to determine the purity and concentration of total RNAs. The generation of complementary DNA (cDNA) was realized by utilizing the First Strand cDNA Synthesis kit (Thermo Scientific). Gene expressions were quantified via the real-time PCR applying the SYBR EX TAQ (Takara, Dalian, China). GAPDH (for mRNA) and U6 (for miRNA) were the internal controls. The calculation of relative expression was on the basis of 2^−ΔΔCt^ method. Primers were used as follows:

LINC00885: 5'-TCCATTGCCCCGTGAGTAAC-3' (forward),
  5'-TGAACCAGCCGGTTACAGTC-3' (reverse);
ZAB2A: 5'-ACTGTATCTCACACTACTAC-3' (forward),
  5'-GAAGGTTAGTGTTATGACTT-3' (reverse);
GAPDH: 5'-CTCTGCTCCTCCTGTTCGAC-3' (forward),
  5'-GCGCCCAATACGACCAAATC-3' (reverse);
U6: 5'-CGTTTTACTTCCTCATACAGCAC-3' (forward),
  5'-GCACCAAGAGACCTGTGACA-3' (reverse).

### Cell proliferation assay

The measurement of cell proliferation was carried out by using reagent CCK-8 (Roche, Basel, Switzerland). CC cells were plated in the 96-well microtiter plates (Corning, NY, USA) with the density of 1 × 10^3^ per well. After incubation for 0, 24, 48, 72, and 96 h, CCK-8 solution (10 μL) was added into each well for 2-h incubation. The absorbance (wavelength: 450 nm) was evaluated to examine the cell viability.

### EdU

The cell proliferation was also examined by utilizing the EdU DNA Proliferation in vitro Detection kit (RiboBio, China). CC cells were seeded into the 96-well plate. Subsequent to transfection for 48 h, CC cells were stained by EdU and the cell nuclei was stained by 4',6-diamidino-2-phenylindole (DAPI) (Cell Signaling Technology, Danvers, MA, USA). The proportion of EdU-positive cells was calculated to determine the cell proliferation.

### Colony formation assay

For colony formation assay, cells were seeded in 6-well plate with 5 × 10^2^ cells each well. After incubation for 2 weeks, the cells were subjected to fixation with 4% paraformaldehyde and staining with 0.1% crystal violet. The colonies with over 50 cells were observed and evaluated with a light microscope.

### TUNEL assay

TUNEL kit (Vazyme, TUNEL Bright-Red Apoptosis Detection Kit, A113) was used to evaluate apoptosis of CC cells. In short, cell slides were blocked by the hydrogen peroxide solution after PBS washing. Subsequent to the permeabilization in Trixon-100 (Sigma-Aldrich Co., St Louis, MO, USA), CC cell slides were incubated with the TUNEL solution for 1.5 h, followed by DAPI (Cell Signaling Technology) staining. The TUNEL-positive cells were observed utilizing the fluorescence microscopy (DMI4000B, Leica, Mannheim, Germany).

### Subcellular fractionation analysis

Subcellular isolation of RNAs in SiHa and HeLa cells was carried out by utilizing the Cytoplasmic and Nuclear RNA Purification Kit (Norgenbiotek Corporation, Thorold, ON, Canada) according to the manufacturer's instructions. The expression of LINC00885 in nucleus and cytoplasm was evaluated by RT-qPCR, with U6 and GAPDH as the nuclear and cytoplasmic controls.

### FISH assay

To determine the cellular localization of LINC00885, FISH was performed by utilizing the RNAscope Multiplex Fluorescent Reagent Kit v2 (Advanced Cell Diagnostics, California, USA). CC cells were subjected to 24-h incubation on the culture slides (Thermo Fisher Scientific, Inc., Waltham, MA, USA), followed by 0.5-h fixation in 4% paraformaldehyde. Then, the cell slides were treated with Hydrogen Peroxide and Protease III, followed by 2-h hybridization with the LINC00885 FISH probe (RiboBio, Guangzhou, China) in the HybEZ Oven (Advanced Cell Diagnostics). Thereafter, cells were washed and subjected to dehydration in the graded ethanol. Finally, slides were stained with DAPI (Cell Signaling Technologies, Danvers, USA). The observation and photographing of the cell slides was accomplished by using a FV1000 confocal laser microscope (Olympus, Tokyo, Japan).

### RIP

The interaction of miR-3150b-3p with LINC00885 and BAZ2A was evaluated by RIP assay using the EZMagna RIP Kit (Millipore, Billerica, MA, USA). CC cells were lysed by using the complete RNA immunoprecipitation (RIP) lysis buffer. The magnetic beads conjugated with the antibodies against Argonaute 2 (Ago2) or immunoglobulin G (IgG) (Millipore, Billerica, MA, USA) were incubated with the CC cell extracts for 6 h at 4℃. After the removal of beads and the elution of RNA, the expressions of miR-3150b-3p, LINC00885, and ZAB2A mRNA were analyzed by RT-qPCR.

### RNA pulldown

The biotin-labeled LINC00885 and antisense LINC00885 (LINC00885-AS) were subjected to reverse transcription by utilizing the Biotin RNA Labeling Mix (Roche, Basel, Switzerland) and T7 RNA polymerase (Takara Biomedical Technology), followed by the treatment of RNase-free DNase I (Roche, Basel, Switzerland) and the purification by RNeasy Mini Kit (Qiagen, MD, USA). Subsequent to the incubation of cell lysates with the biotin-labeled probes and the Dynabeads M-280 Streptavidin (Thermo Fisher Scientific), the RNAs pulled down were eluted and measured by RT-qPCR.

### Luciferase reporter assay

Full sequence of LINC00885 containing the predicted miR-3150b-3p binding sequences or muated miR-3150b-3p binding sequences were respectively cloned into the pmirGLO Expression Vector (Promega, Madison, WI, USA) to generate the LINC00885-WT/MUT reporter plasmids. Wide type BAZ2A 3’UTR with miR-3150b-3p binding site, or with miR-3150b-3p binding site 1/2 mutated was subcloned into pmirGLO vectors to construct BAZ2A-WT, BAZ2A-MUT-1 (with binding site 1 mutated), BAZ2A-MUT-2 (with binding site 2 mutated) and BAZ2A-MUT-1 + 2 (with binding sites 1 and 2 both mutated). These plasmids were co-transfected with miR-3150b-3p mimics or NC mimics respectively into HEK293T cells using the Lipofectamine 2000 (Invitrogen). A dual-Luciferase Reporter Assay System (Promega) was utilized to evaluate the luciferase activities, with Renilla luciferase activity as normalized control.

### Western blot

RIPA protein extraction reagent (Beyotime, Shanghai, China) with the addition of PMSF (Roche, Basel, Switzerland) was used to lyse the cells. Protein extracts were separated by utilizing the 10% sodium dodecyl sulfate polyacrylamide gel electrophoresis (SDS-PAGE), and were then transferred onto the nitrocellulose membranes (Sigma, St. Louis, MO, USA), which were incubated with specific primary antibodies for 12 h, followed by 2-h incubation with secondary antibodies. Detection of protein bands was done by the enhanced chemiluminescent (ECL) method. Primary antibodies against total caspase 3, cleaved-caspase 3, total caspase 9, cleaved-caspase 9, BAZ2A, PCNA, Ki67, and GAPDH were all from Abcam (Cambridge, MA, USA).

### Animal experiments

The five-week-old female athymic BALB/c mice were raised in the pathogen-free condition, and the animal experiment was conducted according to the protocols authorized by the animal center of the First Affiliated Hospital of Nanchang University. All mice were randomly grouped in two, with one group subjected to the subcutaneous injection of SiHa cells transfected with pcDNA3.1 and the other group subjected to the subcutaneous injection of SiHa cells transfected with pcDNA3.1/LINC00885. The SiHa cells with indicated transfection were harvested at a density of 2 × 10^7^ cells/mL. Then, 0.1 mL of the suspend SiHa cells were injected in nude mice at both sides of the posterior flank. Then, tumor volumes were measured every 4 days. At the 20th day after injection, all mice were sacrificed and tumors were dissected for the evaluation of final tumor volume and weight and also for RNA and protein detection via RT-qPCR and western blot analysis.

### Statistical analysis

All experiments were performed for at least 3 times. All data was demonstrated as the mean ± standard deviation (SD). The analyses and presentation of data were conducted by the SPSS 19.0 statistical software and GraphPad Prism V5.0 (GraphPad Software, Inc., La Jolla, CA, USA) software. Student’s t-test or ANOVA were used for the difference measurement between 2 groups or more than 2 groups. The expression correlation was evaluated by Spearman’s correlation analysis. Overall survival of CC patients was analyzed by Kaplan–Meier analysis and log-rank test. *P* < 0.05 was considered to possess the statistical significance.

## Supplementary Information

**Additional file 1**.** Fig. S1**: (A) RT-qPCR analysis was done for detecting the expression levels of three miRNAs (miR-432-5p, miR-4784 and miR-3613-5p) in H8 and four CC cell lines. (B) RT-qPCR analysis was done for measuring the expression of five mRNA candidates of miR-3150b-3p in different cell lines. n.s.: no significance.

## Data Availability

Not applicable.

## Abbreviations

CC: Cervical cancer
lncRNAs: Long non-coding RNAs
LINC00885: Long intergenic non-coding RNA 885
ceRNA: Competitive endogenous RNA
miRNAs: MicroRNAs
3'UTR: 3' Untranslated region
BAZ2A: Bromodomain adjacent to zinc finger domain 2A
NoRC: Nucleolar remodeling complex
RT-qPCR: Reverse transcription-quantitative polymerase chain reaction
EdU: 5-Ethynyl-2′-deoxyuridine
DAPI: 4',6-Diamidino-2-phenylindole
FISH: Fluorescence in situ hybridization
RIP: RNA immunoprecipitation
Ago2: Argonaute 2
SDS-PAGE: Sodium dodecyl sulfate polyacrylamide gel electrophoresis
CESC: Cervical squamous cell carcinoma
