# Supplementary figures and images for: LINC00885 promotes cervical cancer progression through sponging miR-3150b-3p and upregulating BAZ2A
Source: Biol Direct. 2022 Jan 10;17:4. doi: 10.1186/s13062-021-00314-6 (PMC8744347; doi:10.1186/s13062-021-00314-6)

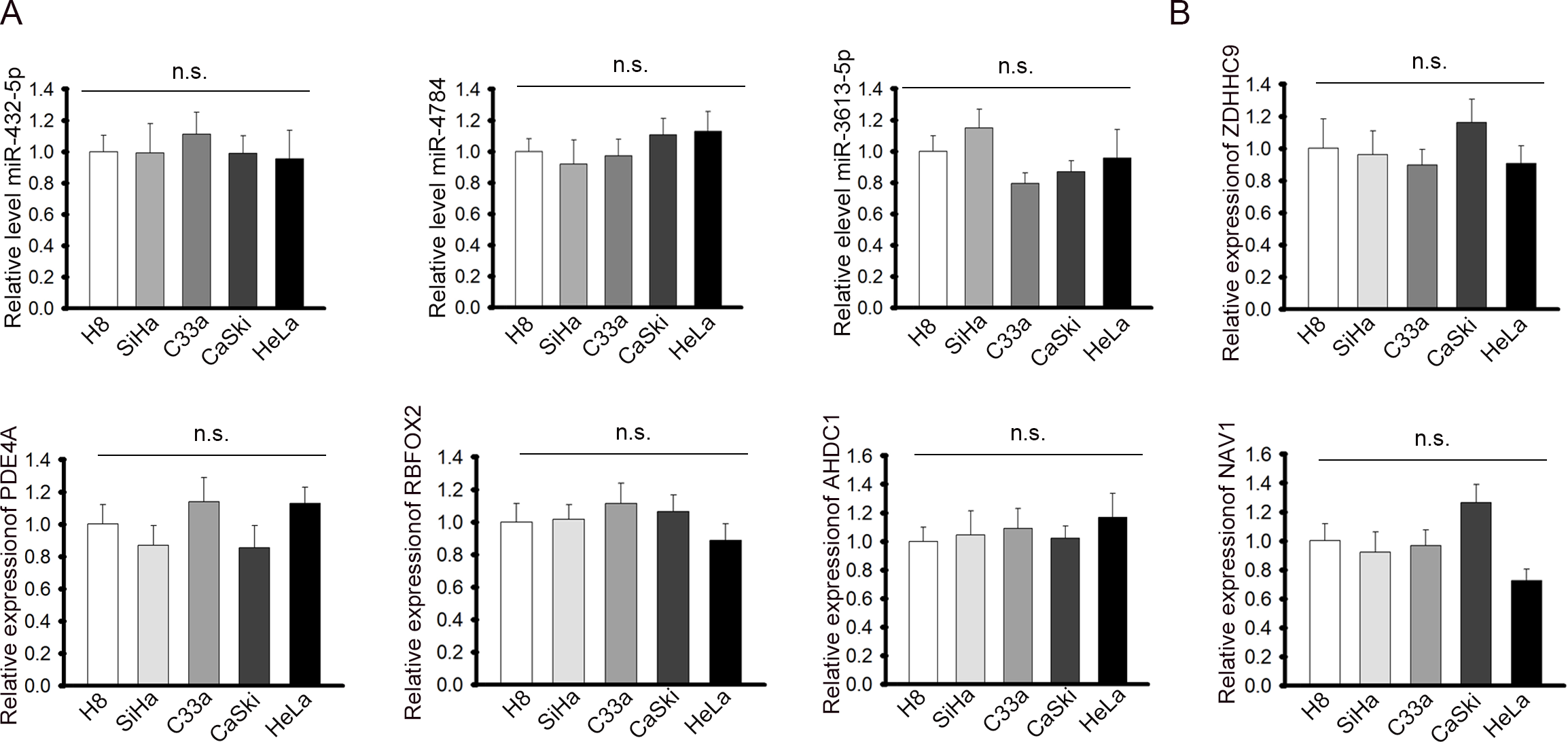

Supplement: Supplementary file 1 — Additional file 1. Fig. S1: (A) RT-qPCR analysis was done for detecting the expression levels of three miRNAs (miR-432-5p, miR-4784 and miR-3613-5p) in H8 and four CC cell lines. (B) RT-qPCR analysis was done for measuring the expression of five mRNA candidates of miR-3150b-3p in different cell lines. n.s.: no significance. [file 13062_2021_314_MOESM1_ESM.tif]
